# Relationships between lead biomarkers and diurnal salivary cortisol indices in pregnant women from Mexico City: a cross-sectional study

**DOI:** 10.1186/1476-069X-13-50

**Published:** 2014-06-10

**Authors:** Joseph M Braun, Rosalind J Wright, Allan C Just, Melinda C Power, Marcela Tamayo y Ortiz, Lourdes Schnaas, Howard Hu, Robert O Wright, Martha Maria Tellez-Rojo

**Affiliations:** 1 Department of Epidemiology, Brown University, 121 S. Main St, Providence, RI 02912, USA; 2 Pediatrics Kravis Children’s Hospital, Departments of Pediatrics and Preventive Medicine, Icahn School of Medicine at Mount Sinai, New York City, New York; 3 The Mindich Child Health & Development Institute, Icahn School of Medicine at Mount Sinai, New York City, New York; 4 Department of Environmental Health, Harvard School of Public Health, Boston, Massachusetts, USA; 5 Department of Epidemiology, Harvard School of Public Health, Boston, Massachusetts, USA; 6 Center for Evaluation Research and Surveys, National Institute of Public Health, Cuernavaca, Morelos, Mexico; 7 Dalla Lana School of Public Health, University of Toronto, Toronto, Ontario, Canada; 8 Division of research on Public Health, National Institute of Perinatology, Mexico City, Mexico

**Keywords:** Lead, Cortisol, Epidemiology, Pregnancy

## Abstract

**Background:**

Lead (Pb) exposure during pregnancy may increase the risk of adverse maternal, infant, or childhood health outcomes by interfering with hypothalamic-pituitary-adrenal-axis function. We examined relationships between maternal blood or bone Pb concentrations and features of diurnal cortisol profiles in 936 pregnant women from Mexico City.

**Methods:**

From 2007–11 we recruited women from hospitals/clinics affiliated with the Mexican Social Security System. Pb was measured in blood (BPb) during the second trimester and in mothers’ tibia and patella 1-month postpartum. We characterized maternal HPA-axis function using 10 timed salivary cortisol measurements collected over 2-days (mean: 19.7, range: 14–35 weeks gestation). We used linear mixed models to examine the relationship between Pb biomarkers and cortisol area under the curve (AUC), awakening response (CAR), and diurnal slope.

**Results:**

After adjustment for confounders, women in the highest quintile of BPb concentrations had a reduced CAR (Ratio: −13%; Confidence Interval [CI]: −24, 1, p-value for trend < 0.05) compared to women in the lowest quintile. Tibia/patella Pb concentrations were not associated with CAR, but diurnal cortisol slopes were suggestively flatter among women in the highest patella Pb quantile compared to women in the lowest quantile (Ratio: 14%; CI: −2, 33). BPb and bone Pb concentrations were not associated with cortisol AUC.

**Conclusions:**

Concurrent blood Pb levels were associated with cortisol awakening response in these pregnant women and this might explain adverse health outcomes associated with Pb. Further research is needed to confirm these results and determine if other environmental chemicals disrupt hypothalamic-pituitary-adrenal-axis function during pregnancy.

## Introduction

The hypothalamic-pituitary-adrenal (HPA) axis is responsible for the regulation of cortisol production in humans. Cortisol plays a major role in neurogenesis and is critical to brain development, especially in memory formation, with the hippocampus being the brain region with the highest concentration of glucocorticoid receptors [1,2]. In humans, cortisol exhibits a diurnal secretory pattern, where levels sharply increase in the first hour after awakening and slowly decline across the day till evening. HPA-axis function can be measured by examining the diurnal pattern of salivary cortisol concentrations and changes in these patterns are associated with health and disease [3]–[6]. Some studies suggest that alterations in maternal HPA-axis function during pregnancy are associated with neurobehavioral deficits [7]–[9].

Gestational Pb exposure is a well-known risk factor for adverse infant, child, and adult behavioral and cognitive development [10,11]. Animal and human studies suggest that lead (Pb) exposure may be associated with alterations in HPA-axis function, where increased Pb exposure is associated with altered adrenocortical response to acute stressors [12]–[16]. Thus, the toxic effects of Pb may be mediated in part through alterations in maternal HPA axis function.

Despite the known role that Pb and cortisol play in brain development, the relationship between Pb and cortisol demonstrated in animals, and potential for maternal HPA-axis disruptions to impact child development, we are not aware of any animal or epidemiological studies that have evaluated associations between Pb exposure and ambulatory diurnal cortisol patterns. Thus, we investigated the relationship between maternal bone or blood Pb levels and salivary cortisol concentrations during pregnancy in a cohort of Mexico City women to determine if cumulative or recent Pb exposure was associated with altered diurnal cortisol profiles or daily cortisol production.

## Methods

### Participants

Participants for this study were enrolled from an ongoing prospective birth cohort in Mexico City. Between July 2007 and February 2011, we invited pregnant women receiving health insurance and prenatal care through the Mexican Social Security System (IMSS) to participate in our study. The IMSS is funded by the federal government, employers, and employees to provide health care to private-sector employees and low- to middle-class workers and their families.

To be eligible for participation in the study, women had to be <20 weeks gestation, ≥18 years old, free of heart or kidney disease, have access to a telephone, plan to reside in Mexico City for the next 3 years, not use steroids (including glucocorticoids) or anti-epilepsy drugs, and not consume alcohol on a daily basis. Institutional review boards at the Harvard School of Public Health, Icahn School of Medicine at Mount Sinai, and Mexican National Institute of Public Health. All women provided informed consent after study protocols were explained to them by research staff.

### Blood Pb measurements

Venous blood was collected in trace metal free tubes from women upon study enrollment during the 2nd trimester, briefly refrigerated at 2-6°C, and subsequently frozen at -20°C until analyzed for Pb using graphite furnace atomic absorption spectrophotometry (Model 3000: Perkin Elmer, Wellesley, MA, USA) [17]. Analysis of blinded quality control samples provided by the Maternal and Child Health Bureau and the Wisconsin State Laboratory of Hygiene Cooperative Blood Lead Proficiency Testing Program demonstrated good precision and accuracy. The limit of detection for this method is <1 µg/dL and the instrument precision is approximately 1 µg/dL. Blood Pb concentrations were examined as continuous variables (µg/dL) and in quintiles.

### Bone Pb measurements

Tibia (cortical bone) and patella (trabecular bone) bone Pb concentration measurements were obtained ~1 month postpartum (mean: 34 days, range: 26–55) using a K-shell X-ray fluorescence instrument [18]. The patella and mid-tibial shaft of each leg were measured for 30 minutes and measures from each leg were averaged by the inverse of the measurement variance. Patella and tibia bone Pb measurements are thought to reflect Pb exposures over the span of decades, with tibia measurements reflecting longer time spans [19]. Bone Pb levels as low as 0.3 µg/g can be quantified using K-XRF; however negative values are sometimes observed when the true bone Pb level is close to 0 [20]. In order to best accommodate these negative values, we created five bone Pb concentration categories where the reference category was all values <2 µg/g and four additional categories were based on quartiles of the values ≥ 2 µg/g. Retaining negative values has been shown to be the least-biased method to accommodate left-censored data, including bone Pb measurements [20,21]. The average imprecision (±SD) of patella and tibia Pb values were 6.4 (±2.3) and 6.9 (±2.3) µg/g, respectively.

### Salivary cortisol measurements

Saliva sampling provides a non-invasive measure of HPA-axis function [3]. Between 14 and 35 weeks gestation (mean: 19.7, standard deviation [SD]: 2.4 weeks), pregnant women were given verbal and written instruction describing when and how to provide five saliva samples each day over two consecutive days during the week or weekend. Before collecting samples, research staff demonstrated what times of the day women should collect their samples and how they should complete their sample collection diary. Women were instructed to provide samples into Salicaps (IBL International, Hamburg, Germany) using the passive drool technique upon awakening, 45 minutes after waking, 4 hours after waking, 10 hours after waking, and at bedtime. Participants were instructed not to eat, brush their teeth, or drink liquids for at least 15 minutes before providing a sample and not drink caffeinated beverages before collecting the first two samples. After sample collection, women recorded the collection time on the tube and in their diary, and were asked to refrigerate samples until pickup, after which they were stored at −70°C until shipment to Dresden, Germany on dry ice.

Saliva samples were assayed in the same batch in duplicate for cortisol using a chemi-luminescence-assay with sensitivity of ~0.16 ng/ml (IBL; Hamburg, Germany, Clemens Kirschbaum). Control sera covering at least three levels of cortisol were run during each 24-hour time period and intra- and interassay coefficients of variation were less than 8%.

Salivary cortisol concentrations exhibit a diurnal rhythm, rising very quickly after waking and then falling throughout the waking hours. We examined total daily cortisol production and two features of women’s diurnal cortisol patterns. The area under the curve (AUC) of the observed salivary cortisol levels is an approximation of total daily cortisol production. It was computed using the trapezoidal rule and standardized to the mean value that all women in the study spent awake each day (~15 hours) [3]. The rapid increase in cortisol concentrations in the morning, known as the cortisol awakening response (CAR) was estimated from the change in cortisol concentrations between the first and second saliva samples of each day. The decline in concentrations over the course of the day is known as diurnal rhythm and was estimated using the change in salivary cortisol concentrations between the 1st and 5th samples of each day.

### Covariates

We considered covariates that might be associated with both maternal Pb exposure and salivary cortisol concentrations [3,22]. Maternal age, marital status, years of education, parity, and smoking status (never, former, and current) was collected using standardized questionnaires. The week cortisol sample collection during pregnancy was calculated based on date of last menstrual period. Weight and height were measured at the baseline study visit and used to calculate body mass index (BMI). We measured calf-circumference with a tape measure at the 1 month postpartum visit and adjusted for it in bone Pb models.

We also collected information on stress or depressive symptoms, although we did not adjust for these in our primary models since this information was collected in the 3rd trimester and these symptoms may be a causal intermediate between Pb exposure and cortisol [23]. Maternal stress was assessed using the Crisis in Family Systems-Revised survey, which inquires about 11 life event domains experienced in the last 6 months (finances, legal problems, career, home events, relationships, safety in the home, safety in the neighborhood, personal medical issues, medical issues with others, prejudice, and authority figures/institutions) [24]. A summary score (range 0 to 11) was calculated by summing the number of domains that women endorsed as having at least one negative life event. We measured depressive symptoms using the Edinburgh Postnatal Depression Scale [25].

### Statistical analyses

Given their right-skewed distribution, all cortisol measures were log_10_-transformed. We used linear mixed models with random intercepts for day and participant, assuming an unstructured covariance matrix to account for the within-woman correlation of cortisol concentrations within and across days [26]. To visualize patterns of cortisol response over the course of a day, we modeled cortisol concentrations as a function of hours since waking using a five-knot restricted cubic polynomial spline [27]. We created smoothed plots according to quantiles of blood and bone Pb concentration to visually assess the impact of Pb on cortisol rhythms.

We report the percent difference in cortisol AUC according to Pb exposure categories. To estimate the association between Pb and CAR, we modeled salivary cortisol concentrations at the first and second samples as a function of time since waking (hours), Pb levels, their interaction, and covariates. We examined the diurnal slope using the first and fifth saliva samples and the same approach used for the CAR. We report the percent change in cortisol per hour according to Pb levels and the beta coefficient of the ratio of these percent changes in cortisol per hour according to Pb levels. We restricted these two analyses to women who reported collecting their samples in the *a prior* defined sampling windows since salivary cortisol concentrations change rapidly in the first hour after waking and incorrectly timed samples can misclassify cortisol profiles (see Additional file 1: Table S1 for *a priori* defined windows) [28].

### Secondary analyses

We conducted a secondary analysis adjusting for 3rd trimester maternal stress and depressive symptoms, the day of the week (weekday vs. weekend), the time of awakening, or number of hours slept on the prior night. We also examined diurnal slopes using the 1st, 3rd, 4th, and 5th salivary cortisol concentrations collected each day. Finally, we re-conducted our tibia and patella Pb analyses after excluding women whose bone Pb measurements imprecision values were >10 (n = 48-53) and 15 (n = 1) µg/g, respectively.

## Results

A total of 1,054 women enrolled in our study during their 2nd trimester of pregnancy. Of these, 937 women provided at least one day of valid cortisol samples. We excluded one woman who had 4 out of the 10 highest cortisol values in the entire cohort. A total of 9,183 saliva samples were provided by the remaining 936 women. With the exception of former-smoking (33.8 vs. 39.8%) and marital status (57.4 vs. 47.5% married), women included in our analyses were similar to the 118 women not included. Our primary blood Pb analyses included 873-918 women with complete data and our bone Pb analyses included 568-594 women with complete data.

On average, women were 28 years of age, modestly educated (mean: 12 years), married (57%), had no children or one child (74%), and never smokers (66%) (Table 1). A total of 199 women (21.3%) had blood Pb concentrations greater than 5 µg/dL and 34 (3.6%) had concentrations greater than 10 µg/dL. Log_10_-transformed blood Pb concentrations were modestly correlated with tibia (Pearson R = 0.26, p < 0.001) and patella (Pearson R = 0.37, p < 0.001) Pb concentrations. Patella and tibia Pb concentrations were modestly correlated as well (Pearson R = 0.38, p < 0.001).

**Table 1:** Univariate description of demographic characteristics, bone/blood Pb concentrations, and cortisol sample characteristics of 936 Mexico City pregnant women (2007–2011)

**Variable**	**N (%)**	**Mean (SD)**	**Min, Max**
Maternal Age		27.8 (5.5)	18.0, 44.0
Maternal Education		11.9 (2.9)	0.0, 21.0
Maternal Weight		64.7 (11.0)	40.0, 108.0
Maternal Height		1.6 (0.1)	1.4, 1.8
Maternal BMI (kg/m^2^)		26.9 (4.2)	17.4, 44.7
Weeks Gestation at Cortisol Collection		19.7 (2.4)	14.1, 35.1
Calf Circumference at 1-Month visit (cm)		33.6 (3.2)	25.0, 47.0
Negative Life Events Score		3.1 (2.1)	0.0, 11.0
Edinburgh Postnatal Depression Scale Score		8.4 (5.6)	0.0, 28.0
Maternal 2nd Trimester Blood Pb Concentration (µg/dL)		3.7 (2.7)	0.5, 23
Maternal Tibia Pb Concentration (µg/g)		2.7 (8.4)	ND, 20
Maternal Patella Pb Concentration (µg/g)		4.6 (8.6)	ND, 43
Waking Time (SD in minutes)		0747 (83)	0305-1300
Hour of Sleep (hours) ^1^		8.3 (1.6)	0.0, 14.5
Marital Status			
Married	537 (57.4)	537 (57.4)	
Free Union	226 (24.1)	226 (24.1)	
Single/Divorced/Separated	173 (18.5)	173 (18.5)	
Number of prior children			
1	356 (38.0)	356 (38.0)	
2	339 (36.2)	339 (36.2)	
3	175 (18.7)	175 (18.7)	
4+	66 (7.1)	66 (7.1)	
Smoking Status During Pregnancy			
Never	614 (65.6)	614 (65.6)	
Former	316 (33.8)	316 (33.8)	
Current	6 (0.6)	6 (0.6)	
Weekday Cortisol Collection^1^			
No	333 (35.6)	333 (35.6)	
Yes	603 (64.4)	603 (64.4)	

ND: Not detectable.

1-On first day of collection.

As expected, salivary cortisol concentrations exhibited a diurnal shape over the course of the day, where they were highest in the hour after awakening, then decreased steeply in the next 5 hours after waking with the slope flattening across the later 10 hours of the day (Additional file 1: Figure S1).

Salivary cortisol AUC concentrations did not vary with blood or bone Pb concentrations (Table 2), although the cortisol AUC was suggestively higher among women in the two highest quantiles of patella Pb.

**Table 2:** Adjusted change in cortisol area under the curve (AUC) according to blood, tibia, and patella Pb concentrations in Mexico City women (2007–2011)

**Pb biomarker/quantile**	**N**	**GM salivary cortisol AUC (nmol-hours)**	**% Difference in cortisol AUC nmol-hours (95% CI)**
**Blood Pb**^**1**^			
Quintile 1 (0- < 1.8 µg/dL)	185	82.0	Ref
Quintile 2 (1.8- < 2.4 µg/dL)	183	88.6	8 (−1, 18)
Quintile 3 (2.4- < 3.4 µg/dL)	184	89.8	9 (0, 19)
Quintile 4 (3.4- < 5.1 µg/dL)	182	88.8	8 (−1, 18)
Quintile 5 (≥5.1 µg/dL)	184	83.8	2 (−6, 12)
Continuous, per 2.5 µg/dL	918		−1 (−4, 1)
**Tibia Pb**^**2**^			
Quantile 1 (<2 µg/g)	266	87.8	Ref
Quantile 2 (2- < 4.3 µg/g)	82	83.2	−5 (−14, 5)
Quantile 3 (4.3- < 6.7 µg/g)	82	89.3	2 (−8, 13)
Quantile 4 (6.7- < 11.1 µg/g)	82	87.5	0 (−10, 10)
Quantile 5 (≥11.1 µg/g)	82	93.4	6 (−4, 18)
**Patella Pb**^**2**^			
Quantile 1 (<2 µg/g)	241	86.6	Ref
Quantile 2 (2- < 4.5 µg/g)	86	87.6	1 (−8, 12)
Quantile 3 (4.5- < 7.8 µg/g)	87	81.8	−6 (−14, 4)
Quantile 4 (7.8- < 12.7 µg/g)	86	97.0	12 (1, 24)
Quantile 5 (>12.7-43.2 µg/g)	87	90.3	4 (−6, 16)

GM: Geometric Mean, AUC: Area Under the Curve.

1-Adjusted for maternal age (continuous years), education (continuous years), body mass index (continuous), marital status (married, free union, or single/separated/divorced), gestational age at time of cortisol collection (continuous weeks), parity (1, 2, 3, or 4+), and maternal smoking status during pregnancy (never, former, or current).

2-Adjusted for above covariates and maternal calf circumference at 1-month postpartum.

**Figure 1:**
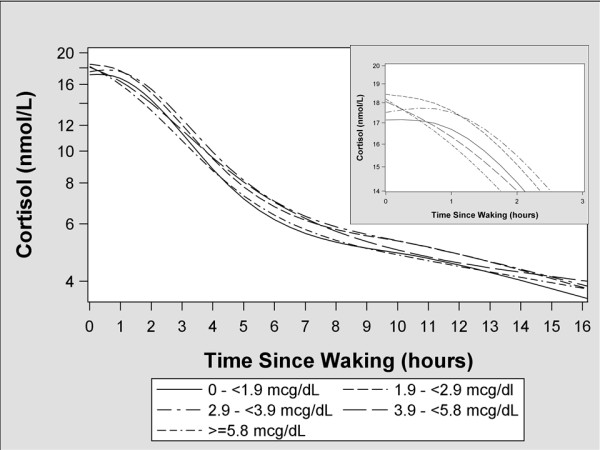
**Adjusted geometric mean salivary cortisol concentrations as a function of time since waking according to 2nd trimester blood Pb quintile**^**1**^**.** 1-Smoothed function was derived by modeling the time since waking as a restricted cubic polynomial spline with 918 women’s salivary cortisol concentrations as the outcome. Only samples collected in the correct time windows are included in this plot. The model was adjusted for maternal age (continuous years), education (continuous years), body mass index (continuous), marital status (married, free union, or single/separated/divorced), gestational age at time of cortisol collection (continuous weeks), parity (1, 2, 3, or 4+), and maternal smoking status during pregnancy (never, former, or current).

**Figure 2:**
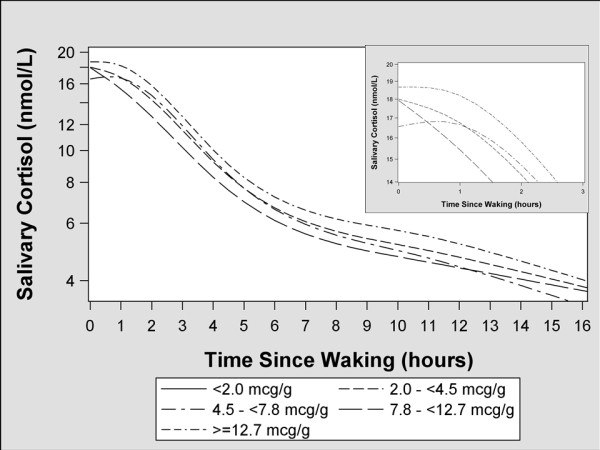
**Adjusted geometric mean salivary cortisol concentrations as a function of time since waking according to 1-month postpartum patella Pb quantile**^**1**^**.** 1-Smoothed function was derived by modeling the time since waking as a restricted cubic polynomial spline with 594 women’s salivary cortisol concentrations as the outcome. Only samples collected in the correct time windows are included in this plot. The model was adjusted for maternal age (continuous years), education (continuous years), body mass index (continuous), marital status (married, free union, or single/separated/divorced), gestational age at time of cortisol collection (continuous weeks), parity (1, 2, 3, or 4+), and maternal smoking status during pregnancy (never, former, or current).

Salivary cortisol concentrations increased 5-7% during the first hour of the day among women in the 1st, 2nd, and 3rd quintiles of blood Pb concentrations, but decreased 5 and 8% among women in the 4th and 5th quintiles, respectively (Table 3 and Figure 1). The CAR among women in the fourth (β: −0.04; 95% confidence interval [CI]: −0.11, 0.02) and fifth (β: −0.06; 95% CI: −0.12, 0.0) quintiles were lower than women in the first quintile. There was not a consistent pattern of relationships between bone Pb concentrations and CAR slopes (Figure 2).

**Table 3:** **Adjusted change in cortisol AM rise according to blood, tibia, and patella Pb concentrations in Mexico City women (2007–2011)**^**1**^

**Pb biomarker/quantile**	**% Change in cortisol per hour (95% CI)**	**β Coefficient (95% CI)**
**Blood Pb (n = 873)**^**2**^		
Quintile 1 (0- < 1.8 µg/dL)	5 (−5, 16)	Ref
Quintile 2 (1.8- < 2.4 µg/dL)	5 (−5, 16)	0.00 (−0.06, 0.06)
Quintile 3 (2.4- < 3.4 µg/dL)	7 (−4, 18)	0.01 (−0.05, 0.07)
Quintile 4 (3.4- < 5.1 µg/dL)	−5 (−14, 5)	−0.04 (−0.11, 0.02)
Quintile 5 (≥5.1 µg/dL)	−8 (−16, 2)	−0.06 (−0.12, 0.00)
Continuous, per 2.5 µg/dL	N/A	−0.02 (−0.04, 0.00)
**Tibia Pb (n = 575)**^**3**^		
Quantile 1 (<2 µg/g)	4 (−4, 12)	Ref
Quantile 2 (2- < 4.3 µg/g)	−8 (−21, 7)	−0.05 (−0.12, 0.03)
Quantile 3 (4.3- < 6.7 µg/g)	7 (−7, 23)	0.02 (−0.05, 0.09)
Quantile 4 (6.7- < 11.1 µg/g)	−9 (−22, 5)	−0.05 (−0.12, 0.02)
Quantile 5 (≥11.1 µg/g)	8 (−7, 25)	0.02 (−0.05, 0.09)
**Patella Pb (n = 568)**^**3**^		
Quantile 1 (<2 µg/g)	2 (−6, 10)	Ref
Quantile 2 (2- < 4.5 µg/g)	11 (−3, 27)	0.04 (−0.03, 0.11)
Quantile 3 (4.5- < 7.8 µg/g)	−11 (−22, 3)	−0.05 (−0.12, 0.02)
Quantile 4 (7.8- < 12.7 µg/g)	−2 (−15, 12)	−0.01 (−0.08, 0.06)
Quantile 5 (>12.7-43.2 µg/g)	7 (−7, 23)	0.03 (−0.04, 0.10)

1-Only includes women who collected their first and second cortisol samples in the correct time window.

2-Salivary cortisol concentrations are modeled as a function of time since waking, Pb biomarker, a Pb biomarker x time interaction term, and covariates.

3-Adjusted for above covariates and maternal calf circumference at 1-month postpartum.

Diurnal cortisol slopes were not different across quintiles of blood Pb concentrations (Table 4 and Figure 1). Women in the top quantile of tibia Pb concentrations had flatter diurnal slopes (74% decline per 15 hours) than women with tibia Pb concentration < 2 µg/g (77% decline per 15 hours), but the 95% confidence interval (CI) for this difference included the null value (β: 0.04; CI: −0.03, 0.11). A similar pattern was seen when comparing women in the top (−73% decline per 15 hours) and bottom (−77% decline per 15 hours) quantiles of patella Pb concentrations (β: 0.06; CI: −0.01, 0.12), but this difference did not reach conventional levels of statistical significance.

**Table 4:** **Adjusted change in cortisol diurnal slope according to blood, tibia, and patella Pb concentrations in Mexico City women from 2007-2011**^**1**^

**Pb biomarker/quantile**	**% Change in cortisol per 15 hours (95% CI)**	**β Coefficient (95% CI)**
**Blood Pb (n = 918)**^**2**^		
Quintile 1 (0- < 1.8 µg/dL)	−77 (−79, −75)	Ref
Quintile 2 (1.8- < 2.4 µg/dL)	−77 (−79, −75)	0.00 (−0.06, 0.05)
Quintile 3 (2.4- < 3.4 µg/dL)	−76 (−78, −73)	0.03 (−0.03, 0.08)
Quintile 4 (3.4- < 5.1 µg/dL)	−76 (−78, −73)	0.02 (−0.03, 0.08)
Quintile 5 (≥5.1 µg/dL)	−78 (−79, −75)	−0.01 (−0.06, 0.05)
Continuous, per 2.5 µg/dL	N/A	0.00 (−0.02, 0.02)
**Tibia Pb (n = 594)**^**3**^		
Quantile 1 (<2 µg/g)	−77 (−79, −76)	Ref
Quantile 2 (2- < 4.3 µg/g)	−79 (−82, −76)	−0.05 (−0.11, 0.02)
Quantile 3 (4.3- < 6.7 µg/g)	−77 (−79, −73)	0.00 (−0.07, 0.07)
Quantile 4 (6.7- < 11.1 µg/g)	−77 (−80, −74)	−0.01 (−0.08, 0.06)
Quantile 5 (≥11.1 µg/g)	−74 (−78, −71)	0.04 (−0.03, 0.11)
**Patella Pb (n = 587)**^**3**^		
Quantile 1 (<2 µg/g)	−78 (−79, −76)	Ref
Quantile 2 (2- < 4.5 µg/g)	−77 (−80, −74)	−0.01 (−0.08, 0.05)
Quantile 3 (4.5- < 7.8 µg/g)	−77 (−80, −74)	−0.01 (−0.08, 0.05)
Quantile 4 (7.8- < 12.7 µg/g)	−79 (−81, −76)	−0.04 (−0.11, 0.03)
Quantile 5 (>12.7-43.2 µg/g)	−73 (−76, −69)	0.06 (−0.01, 0.12)

1-Only includes women who collected their first and second cortisol samples in the correct time window.

2-Salivary cortisol concentrations are modeled as a function of time since waking, Pb biomarker, a Pb biomarker x time interaction term, and covariates.

3-Adjusted for above covariates and maternal calf circumference at 1-month postpartum.

### Secondary analyses

Adjusting for 3rd trimester stress or depressive symptoms, time of waking, and hours of sleep did not substantively change the pattern of most of our results (subset of 709 women). The pattern of results for diurnal slopes was similar regardless of whether we used 2 or 5 samples to characterize the diurnal slope. Our results were not substantively changed when we excluded women with relatively imprecise bone Pb measurements (results not shown).

## Discussion

Among pregnant women from Mexico City, second trimester salivary cortisol concentrations followed the expected diurnal pattern, demonstrating that our study protocol was successful in capturing cortisol daily rhythms. Characteristics of this diurnal pattern differed according to blood and bone Pb concentrations after adjusting for demographic and anthropometric characteristics suggesting that Pb exposure could alter cortisol rhythms. However, associations between bone Pb concentrations and diurnal cortisol rhythms did not reach convention levels of statistical significance.

The use of multiple Pb exposure biomarkers allowed us to finely dissect this relationship with respect to different windows of exposure. The association between Pb exposure and salivary cortisol rhythms depended on the Pb exposure measurement matrix. This is intriguing given that these two matrices represent different sources and timing of Pb exposure. Blood Pb levels are correlated with the pool of biologically active Pb and represent recently ingested or inhaled Pb, as well as Pb released from the skeleton [19]. In contrast, bone Pb represents past Pb exposures, including those from decades ago, that have been deposited into hydroxyapatite crystal [3]. Consistent with these two matrices representing different periods and sources of exposure, we observed modest correlations between bone and blood Pb concentrations. We speculate that past or concurrent Pb exposures – represented by bone and blood Pb levels, respectively – could have different effects on HPA-axis function.

Specific periods of vulnerability have been identified in prior studies of Pb exposure and child cognition [29], but little is known about the potential associations between Pb exposures at different periods of life and HPA-axis function. Pb exposures earlier in pregnancy or before pregnancy could impact maternal HPA-axis function and future studies should consider additional windows of vulnerability (e.g., 1st trimester). Alternatively, specific pattern of associations could result from pregnancy-induced changes in bone Pb mobilization, bone turnover, and HPA-axis function [30]–[32].

We are not aware of other studies examining the relationship between environmental chemical exposures and daily diurnal cortisol patterns in any human populations. Two previous studies have examined the relationship between blood/bone Pb concentrations and the response to an acute adrenocortical stressor response in children and adults [12,13]. Gump et al. found that elevated gestational and childhood blood Pb concentrations were associated with heightened cortisol responses in children following an acute stressor task [12]. Fortin and colleagues found the opposite association in a group of occupationally exposed men [13]. In their study, bone and blood Pb concentrations were associated with increased adrenocorticotropic hormone (ACTH) concentrations and decreased cortisol concentrations before the application of an acute stressor and increased ACTH: cortisol ratios after stressor application, suggesting that Pb exposure may be associated with adrenal hypo-responsiveness to pituitary stimulation. Consistent with this, experimental studies in rats suggest that Pb exposure is associated with elevated corticosterone concentrations during pregnancy [16]. Studies of adult rodents found that Pb exposure was associated with elevated ACTH and corticosterone concentrations, as well as increased anxiety-like behaviors [14].

The presented results and prior epidemiological studies illustrate potentially important differences in HPA-axis function response to Pb exposure that may depend on the choice of measure for HPA-axis function (e.g., acute stressor vs. diurnal rhythm) and timing of Pb exposure. The latter is important for fetal development since disruption of homeostatic cortisol function during the sensitive prenatal period may have profound effects later in life since the developing fetus is more sensitive to environmental exposures [33]. Changes in maternal cortisol levels during gestation could alter placental expression of enzymes involved in glucocorticoid signaling and metabolism, ultimately leading to altered neurobehavior [8,9].

Both gestational Pb and stress exposure may increase the risk of poorer mental and physical development, reduced IQ, and delinquent behavior in children [7,10,34,35]. The neurotoxic mechanisms of Pb exposure are diverse and may be outcome dependent and the association between Pb and HPA-axis function has been relatively unstudied as a potential mechanism of Pb toxicity [36]. A prior study found that higher maternal 3rd trimester serum cortisol levels were associated with lower child IQ [7]. While this finding may seem discordant with our finding of reduced CAR among women with elevated blood Pb levels, the cortisol measures in this and the prior study reflect different features of HPA-axis function. The CAR is the change in cortisol over the first hour of the day, while a single serum measurement assesses cortisol concentrations at one time during the day. Additional studies will need to address the relationships between Pb, diurnal cortisol rhythms, and child neurodevelopment to determine if alterations in HPA-axis function are a mediator of Pb-induced cognitive deficits. Future studies should also identify which patterns of maternal diurnal cortisol rhythms during pregnancy are associated with adverse child neurodevelopment.

While salivary cortisol concentrations are a non-invasive method of measuring HPA-axis function, there are sources of variability that may impact the accuracy and precision of our results. First, cortisol exhibits both within-day and within-person variability that we attempted to reduce and account for the by collecting 10 timed samples across two days and using appropriate statistical models. Second, accurate estimation of the CAR and diurnal slope requires that the time of saliva sample collection be reported correctly. Seventy-percent of women who reported collecting their cortisol samples in the acceptable time windows had at least one day with a positive CAR. This is similar to prior studies reporting that 75% of persons have increasing cortisol concentrations after awakening [37]. Incorrectly reported times of waking or 1st and 2nd sample collection could produce a negative CAR that is not due to biological variation, but measurement error. This could systematically bias our results if socioeconomic factors associated with Pb exposure are also associated with protocol adherence; however, we have no reason to suspect this is the case [38].

Residual confounding may have produced the pattern of results we observed. For instance, women with more Pb exposure may live in poorer neighborhoods with higher crime, resulting in changes in the pattern of salivary cortisol concentrations [39]. This seems unlikely given that the pattern of our results was similar when we adjusted for negative life events (including neighborhood safety) and depressive symptoms. Additional confounding may also arise because pregnancy-induced changes in metabolism may alter cortisol concentrations and increase blood Pb levels by releasing bone Pb stores through bone resorption.

## Conclusions

Cortisol awakening response was associated with increased blood Pb concentrations among pregnant women in this cohort. While many studies of endocrine disruption have focused on thyroid and gonadal hormones, the association between other environmental chemicals and cortisol homeostasis remains unstudied in humans. The importance of glucocorticoids in brain development and sensitivity of salivary cortisol levels to a known toxicant illustrates the importance of understanding how environmental chemicals might impact both cortisol rhythms and subsequent child health and development.

## Abbreviations

ACTH: Adrenocorticotropic Hormone; AUC: Area Under the Curve; BMI: Body Mass Index; CAR: Cortisol Awakening Response; CI: 95% Confidence Interval; HPA: Hypothalamic-Pituitary-Adrenal; IMSS: Mexican Social Security System; Pb: Lead; SD: Standard Deviation.

## Competing interest

Dr. Braun was financially compensated for conducting a re-analysis of the international pooled study of lead exposure for the plaintiffs in a public nuisance case. The other authors have no conflicts of interest.

## Authors’ contributions

JB analyzed the data, performed the literature review, and drafted the manuscript. LS, MMT-R, and RW designed the cohort, reviewed the manuscript, and were responsible designing the original cohort and for obtaining funding. MTO reviewed the manuscript and assisted in collected saliva samples from women. MP and AJ reviewed the manuscript and provided statistical support. RW provided intellectual feedback on interpreting salivary cortisol levels and reviewed the manuscript. HH provided feedback and interpretation of bone lead measurements and reviewed the manuscript. All authors read and approved the final manuscript.

## Authors’ information

Robert O Wright and Martha Maria Tellez-Rojo are senior authors.

## Supplementary Material

Additional file 1 **Relationships between lead biomarkers and diurnal salivary cortisol indices in pregnant women from Mexico City.** Supplemental **Table 1.** Number of Saliva Samples Collected Inside and Outside Acceptable Time Windows Among 936 Pregnant Women From Mexico City (2007-2011). Supplementary **Figure 1.** Smoothed Geometric Mean Salivary Cortisol Concentrations as a Function of Time Since Waking Among Pregnant Mexico City Women (2007-2011) With Samples Collected in the Correct Time Window. Click here for file
