# Supplementary material for: Relationships between lead biomarkers and diurnal salivary cortisol indices in pregnant women from Mexico City: a cross-sectional study
Source: Environ Health. 2014 Jun 10;13:50. doi: 10.1186/1476-069X-13-50 (PMC4068833; doi:10.1186/1476-069X-13-50)
Supplement: Additional file 1 — Relationships between lead biomarkers and diurnal salivary cortisol indices in pregnant women from Mexico City. Supplemental Table 1. Number of Saliva Samples Collected Inside and Outside Acceptable Time Windows Among 936 Pregnant Women From Mexico City (2007-2011). Supplementary Figure 1. Smoothed Geometric Mean Salivary Cortisol Concentrations as a Function of Time Since Waking Among Pregnant Mexico City Women (2007-2011) With Samples Collected in the Correct Time Window. [file 1476-069X-13-50-S1.docx]

**Supplementary Material**

**Title:** Relationships between lead biomarkers and diurnal salivary cortisol indices in pregnant women from Mexico City

**Authors:** Joseph M. Braun, Rosalind J. Wright, Allan C. Just, Melinda C. Power, Marcela Tamayo Ortiz, Lourdes Schnaas, Howard Hu, Robert O. Wright, and Mara Tellez-Rojo

**Table of Contents:**

Supplemental Table 1: Number of Saliva Samples Collected Inside and Outside Acceptable Time Windows Among 936 Pregnant Women From Mexico City (2007-2011)

Supplementary Figure 1: Smoothed Geometric Mean Salivary Cortisol Concentrations as a Function of Time Since Waking Among Pregnant Mexico City Women (2007-2011) With Samples Collected in the Correct Time Window

Supplemental Table 1: Number of Saliva Samples Collected Inside and Outside Acceptable Time Windows Among 936 Pregnant Women From Mexico City (2007-2011)

| Saliva Sample | Time Window (hours)^1^ | Mean Hours Since Waking (SD) | Before Window (Row %) | Inside Window (Row %) | After Window (Row %) | Cortisol GM nmol/L (GSD) |
| --- | --- | --- | --- | --- | --- | --- |
| Sample 1 | 0 to 0.25 | 0.1 (0.5) | 0 (0.0) | 1629 (88.3) | 216 (11.7) | 17.6 (1.8) |
| Sample 2 | 0.5 to 1.0 | 1.0 (0.6) | 4 (0.2) | 1438 (78.0) | 401 (21.8) | 16.9 (1.9) |
| Sample 3 | 3.0 to 6.5 | 4.5 (1.0) | 10 (0.5) | 1751 (95.4) | 74 (4.0) | 8.5 (1.8) |
| Sample 4 | 7.5 to 11.5 | 10.7 (1.4) | 3 (0.2) | 1540 (84.0) | 291 (15.9) | 5.0 (2.0) |
| Sample 5 | 11.5 to 20.0 | 15.2 (1.6) | 10 (0.5) | 1817 (99.0) | 9 (0.5) | 3.9 (2.1) |

GM: Geometric Mean, GSD: Geometric Standard Deviation,

1-The time windows that women were asked to collect samples in.

Supplementary Figure 1: Smoothed Geometric Mean Salivary Cortisol Concentrations as a Function of Time Since Waking Among Pregnant Mexico City Women (2007-2011) With Samples Collected in the Correct Time Window


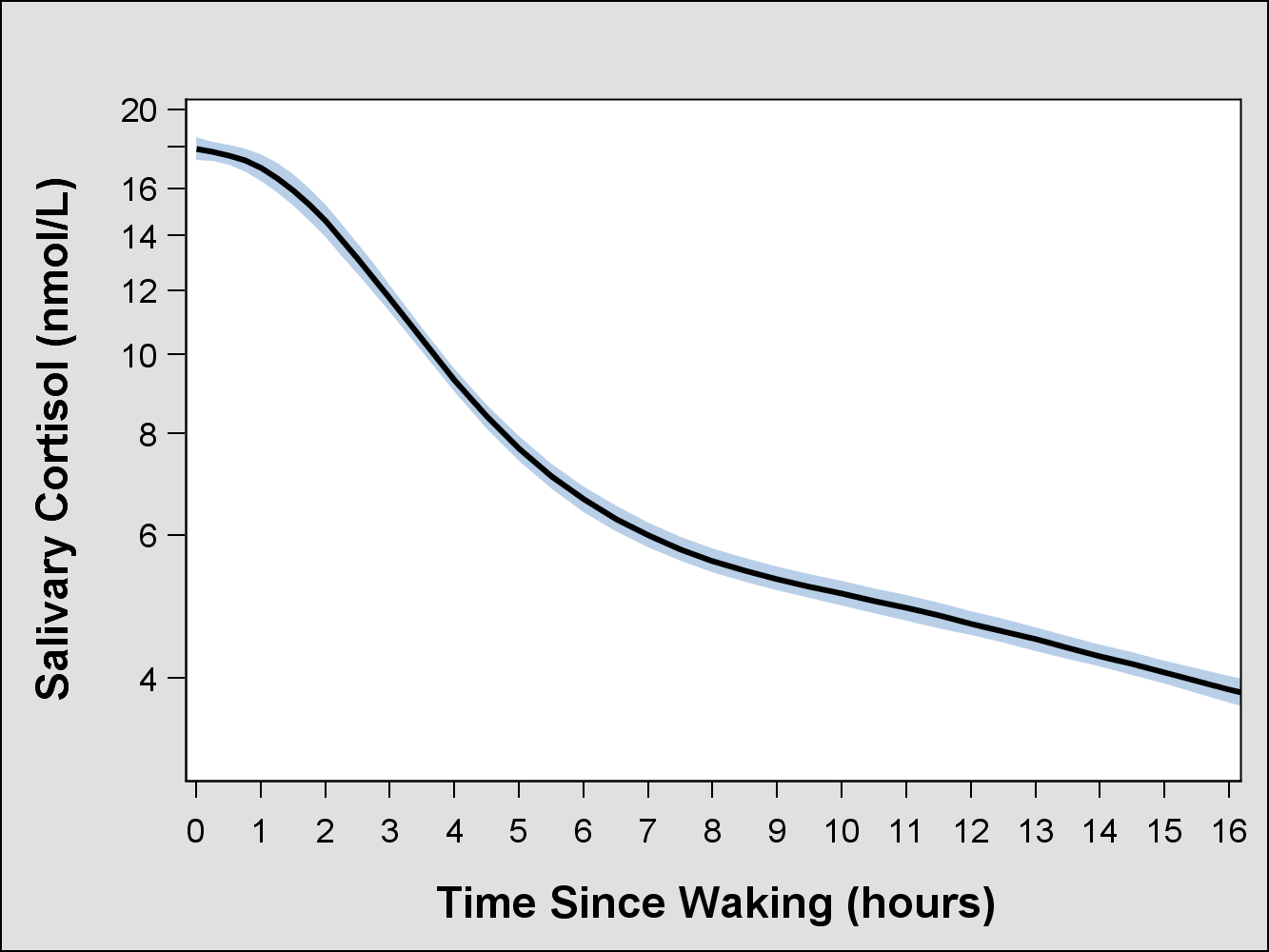


*-Smoothed function was derived by modeling the time since waking as a restricted cubic polynomial spline with 936 women’s salivary cortisol concentrations as the outcome. Only samples collected in the correct time windows are included in this plot.
